# Germline Pathogenic Variant in the APC Gene Suggestive of Gardner Syndrome in a Pony

**DOI:** 10.1155/crve/1395580

**Published:** 2026-04-24

**Authors:** J. Eric Martin, Silke Hecht, Linden Craig, Sian A. Durward-Akhurst, Jillian L. Marlowe, Melissa T. Hines

**Affiliations:** ^1^ University of Tennessee College of Veterinary Medicine, Knoxville, Tennessee, USA, tennessee.edu; ^2^ Department of Veterinary Clinical Sciences, University of Minnesota, St. Paul, Minnesota, USA, umn.edu

## Abstract

A 12‐year‐old pony mare was presented for evaluation of dental disease and nasal discharge. At presentation, clinical signs included bilateral nasal discharge, cutaneous masses, and numerous hard enlargements involving the bones of the skull, maxilla, mandible, and cervical vertebrae. Oral exam revealed advanced dental disease with hard enlargements adjacent to and between numerous cheek teeth. Radiographs and computed tomography confirmed the presence of severe dental disease and proliferative bone lesions disseminated along the skull, hyoid apparatus, and cranial cervical vertebrae. The bony proliferations extended into the subcutis, nasal cavity, paranasal sinuses, orbits, cranial vault, and vertebral canal. Multifocal osteomas were considered the primary differential, and this was confirmed on biopsy of a bony mass. Due to the extent of the lesions and deterioration of the patient’s quality of life, euthanasia was elected. On necropsy, multiple osteomas were present on the skull and to a lesser extent the cervical vertebrae. Additional abnormalities included multiple mucosal polyps in the small intestine, epidermal inclusion cysts, and adrenocortical adenomas. These findings resemble those seen in Gardner syndrome, a hereditary disease of people characterized by gastrointestinal polyps and extraintestinal manifestations, including osteomas, epidermoid cysts, adrenal tumors, and dental abnormalities. Historically, Gardner syndrome in people has been considered a separate condition from familial adenomatous polyposis (FAP). Gardner syndrome is now considered a variant of FAP associated with mutations in the adenomatous polyposis coli (APC) gene. Whole genome sequencing and variant discovery in the pony identified multiple unique variants, including a likely pathogenic single base pair insertion leading to a frameshift in APC (ENSECAP00000007276.1:p.Glu1527ArgfsTer9). While osteomas have been infrequently reported in horses, to the authors’ knowledge, there have been no cases of Gardner syndrome described in equids. This case is highly suggestive of Gardner‐like syndrome in an equid.

## 1. Introduction

Gardner syndrome, also known as Gardner’s syndrome, is a genetic condition characterized by gastrointestinal polyps and several extracolonic manifestations, with osteomas and dental abnormalities being common [1–4]. To date, the condition has not been reported in species other than human beings. Though historically Gardner syndrome had been considered a separate genetic condition from familial adenomatous polyposis (FAP), currently, it is considered a variant or subtype of the condition existing on a spectrum of clinical manifestations of FAP [3]. FAP is a rare genetic disorder characterized by adenomatous polyps within the colorectal region that progresses to colorectal carcinoma in most patients [4]. In addition to the colonic manifestations, Gardner syndrome is characterized specifically by osteomas, dental abnormalities, epidermoid cysts, desmoid tumors, and congenital hypertrophy of the retinoid epithelium (CHRPE) [1–4]. FAP is an autosomal dominant disorder arising from mutations within the adenomatous polyposis coli (APC) gene on Chromosome 5q21‐22 [5–9]. The APC gene produces the APC protein, which is a tumor suppressor protein involved in multiple cellular functions, including the control of cell growth via regulation of the timing of cell cycles [5–9]. The specific mutation within the APC gene influences the severity of the gastrointestinal lesions as well as the specific extracolonic manifestations, resulting in a spectrum of clinical variants or subtypes of FAP such as Gardner and Turcot syndromes [1, 10–13]. In human patients, FAP is of particular importance due to its association with colorectal cancer.

In this case, a 12‐year‐old pony mare was presented for nasal discharge that had been refractory to treatment by the referring veterinarian as well as concerns regarding the dentition of the patient. A physical exam combined with an oral exam revealed unusual clinical findings, including multiple firm enlargements involving the skull and cervical spine as well as advanced dental disease associated with hard enlargements within interproximal spaces. The extent of the lesions suggested a unique disease process. This case report outlines the diagnostic evaluation of this patient, including the clinical presentation, advanced imaging, necropsy findings, and histopathology results as well as genetic sequencing. The findings support the suspicion of Gardner‐like syndrome in this pony.

## 2. Case Presentation

### 2.1. History and Clinical Presentation

A 12‐year‐old pony mare was referred for evaluation of advanced dental disease and nasal discharge. The pony had been acquired by a rescue organization approximately 4 months prior to presentation with a body condition score (BCS) of 1/9 (Henneke system). The patient was observed to quid during the mastication of a senior feed and hay. Copious malodorous purulent nasal discharge was present bilaterally. The referring veterinarian found extensive dental disease on oral examination, and a tentative diagnosis of advanced periodontal disease with secondary sinusitis was made. The patient was treated with odontoplasty, dietary adjustment to a soaked pelleted senior feed, and multiple courses of antibiotics and anti‐inflammatories. While some improvement in the BCS was seen, only a mild transient improvement was noted in the nasal discharge.

Upon presentation to the University of Tennessee Veterinary Medical Center, the pony was bright, alert, and responsive. The temperature and heart rate were within normal limits while the respiratory rate (RR) was slightly elevated (*T* = 100.7°F, *P* = 48/min, RR = 36/min). The pony weighed 174 kg with a BCS of 4/9. Facial asymmetry was noted upon visual exam, and malodorous mucopurulent nasal discharge was present bilaterally. Palpation of the head and jawline revealed numerous hard irregular 1–3‐cm‐diameter enlargements along the entire mandible as well as the entire head and face. The patient did not exhibit evidence of discomfort upon palpation of the skull. Additional firm enlargements were noted in association with the cervical spine. Several firm cutaneous nodules were also on the neck and thorax.

**Figure 1:**
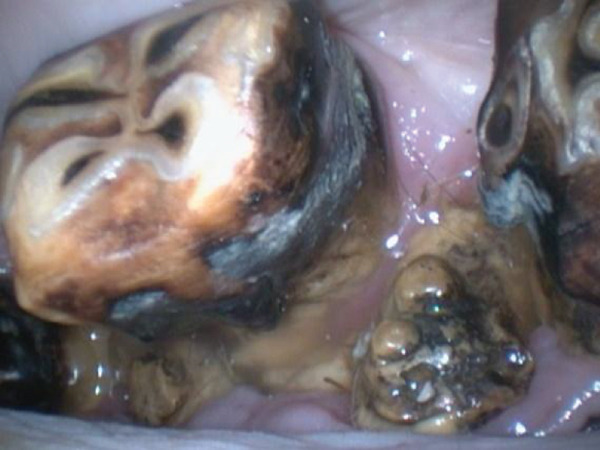
Hard enlargement within the interproximal space of 409 and 410.

An oral examination was performed under sedation. Halitosis was appreciated after application of a full‐mouth speculum. On oral exam, it was noted that odontoplasty had been previously performed on all four arcades save the incisors, which was consistent with the history. In addition, numerous diastemata of varying degrees existed between the cheek teeth, and advanced periodontal disease was present due to the presence of putrefied feedstuff interproximally. Numerous cheek teeth were displaced and/or misaligned. In addition, all cheek teeth were discolored due to oxidative staining of the clinical crown, which was assumed to be secondary to the patient’s inability to properly masticate feedstuff, therefore compromising the natural cleansing process of the oral cavity. Of note was the presence of bony enlargements within numerous interproximal spaces (Figure 1). The growths appeared to contribute to the migration of numerous teeth from their normal position as well as the periodontal disease.

Bloodwork was submitted for CBC, fibrinogen, chemistry, and serum amyloid A. Only mild abnormalities were noted, including a leukopenia characterized by neutropenia (WBC = 4.4 × 10^3^/*μ*L, reference range 4.6–12 × 10^3^/*μ*L; neutrophils = 1.65 × 10^3^/*μ*L, reference range 2.6–5.5 × 10^3^/*μ*L), decreased creatinine (0.6 mg/dL, reference range 0.82–2.1 mg/dL), decreased albumin (2.7 g/dL, reference range 2.8–3.6 g/dL), elevated glucose (178 mg/dL, reference range 76.2–123 mg/dL), and decreased total bilirubin (0.3 mg/dL, reference range 0.6–2.1 mg/dL).

### 2.2. Diagnostic Investigations/Imaging

**Figure 2:**
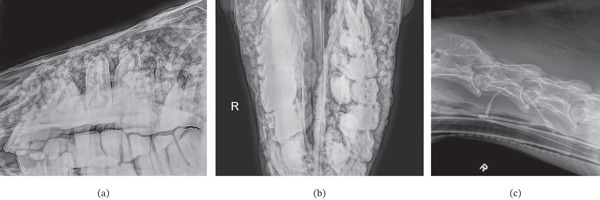
(a–c) Lateral (a) and dorsoventral (b) radiographs of the head and lateral radiograph of the cervical spine (c). There are numerous variable‐sized rounded mineral nodules associated with the osseous surfaces of the skull, plane of the nasal cavities (especially right‐sided), and cervical vertebrae. There is also evidence of severe multifocal dental disease with malocclusion, wave mouth, and tooth displacement. Abbreviation: R, right.

Lateral and dorsoventral radiographs of the head and lateral radiographs of the cervical spine were acquired. There were numerous variable‐sized rounded mineral nodules associated with the osseous surfaces of the skull, plane of the nasal cavities (especially right‐sided), and cervical vertebrae (Figure 2a–c). There was also evidence of severe multifocal dental disease with malocclusion, wave mouth, tooth displacement, and suspected osteomyelitis of the left mandible.

**Figure 3:**
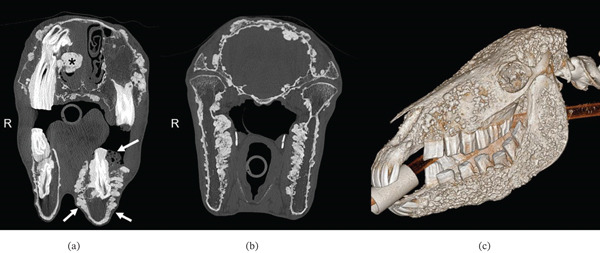
(a–c) Transverse CT images at the level of the nasal cavity (a) and the temporomandibular joints (b) and a surface volume–rendered 3D reconstruction of the CT image dataset (c). Note the extensive multifocal variable‐sized periosteal and endosteal proliferations associated with the osseous structures. The largest lesion is associated with the right nasal cavity (∗ in a), which additionally contains a large amount of soft tissue– or fluid‐attenuating material. Note also the dental misalignment/malocclusion, asymmetry of the dental arcades, and expansion of the left mandible with soft tissue emphysema (arrows in a). Abbreviation: R, right.

Computed tomography (CT) was performed for further evaluation (Figure 3a–c). Transverse images were acquired from the nasal planum through the second cervical vertebra before and after intravenous administration of iodinated contrast medium. Disseminated along the osseous margins of the skull (bones of the neurocranium and viscerocranium, nasal turbinates, and mandible), the hyoid apparatus, and the cranial cervical vertebrae, there were numerous proliferative bone lesions, ranging from submillimeter size to 2.4 cm. The largest lesion was associated with the right nasal cavity. These bony proliferations affected the periosteal as well as endosteal surfaces and protruded into the subcutaneous tissues, nasal cavity, paranasal sinuses, bilateral orbits, cranial vault, and vertebral canal. Lesions were smoothly marginated, and there was no evidence of associated lysis of osseous structures. The skull was most severely affected by these new bone formations. Based on the benign appearance of the lesions, multifocal osteomas were primarily considered.

There was multifocal dental disease with severe malocclusion and undulant margins to the occlusal surfaces (“wave mouth”), marked lateral angulation of the left third mandibular premolar (307) and the left first mandibular molar (309) teeth, emphysema surrounding the mandibular cheek teeth, severe widening of the alveoli, and multifocal mandibular lysis indicative of osteomyelitis. The possibility of osseous proliferative lesions being at least partially responsible for dental malalignment was considered likely.

In addition to containing numerous bony proliferative lesions as described above, the right nasal cavity and right paranasal sinuses were nearly completely filled with fluid‐ or soft tissue–attenuating material, suggesting upper airway obstruction by the osseous proliferative lesions.

Biopsies were taken from a cutaneous nodule on the right neck and a bony mass on the right maxilla. The cutaneous nodule was an infundibular cyst, and the bony lesion was consistent with an osteoma. It was at this point that the combination of clinical signs, imaging, and biopsy findings suggested the possibility of Gardner‐like syndrome. Due to the poor prognosis and deterioration of the patient’s quality of life, euthanasia was elected, and a necropsy was performed.

### 2.3. Necropsy Findings

On external exam, there was bilateral pasty tan nasal discharge and two skin nodules: one on the left side of the crest of the neck (13 × 11 × 8 mm) and one on the right side of the crest of the neck (9 × 7 × 4 mm). Microscopically, these were both epidermal inclusion cysts.

**Figure 4:**
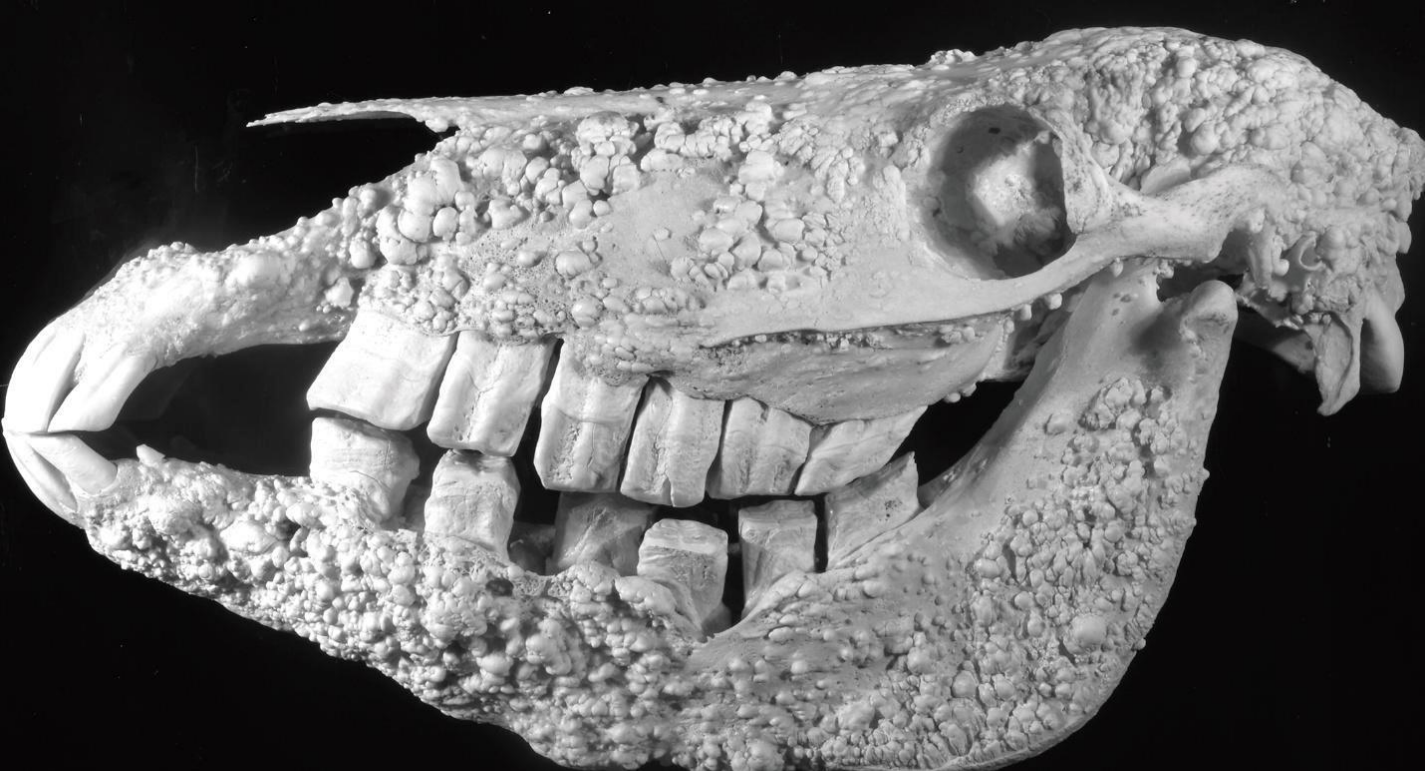
The skull covered in bony nodules.

The skull and to a lesser extent the cervical vertebrae had dozens of 2‐mm‐to‐2‐cm hard irregular bony nodules (Figure 4). The mandible and maxilla were most affected, but nodules were also present within the calvarium where they caused depressions in the cerebrum. The bony nodules within the nasal cavity and sinuses resulted in obstruction and impaction of gray–green pasty malodorous material. Microscopically, the bony nodules consisted of dense compact lamellar bone. These findings were consistent with a diagnosis of multifocal endosteal and periosteal osteomas.

**Figure 5:**
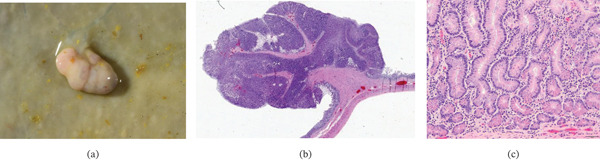
A pedunculated mucosal polyp in the small intestine. Dozens of similar polyps were present in the small intestine. (a) Gross appearance. (b) Subgross showing the uniformly adenomatous nature of the polyp. (c) Photomicrograph of the crypts within the polyp lined by well‐differentiated columnar cells with Paneth cells at the base of the crypts (hematoxylin and eosin, 200x magnification).

Both adrenal glands had multiple adrenocortical adenomas. The pituitary gland was normal grossly and microscopically. The small intestine had dozens of sessile 1–2‐mm mucosal polyps and pedunculated mucosal polyps up to 15 mm (Figure 5a,b) that microscopically consisted of tortuous crypts lined by a single layer of well‐differentiated columnar epithelium with basal nuclei and Paneth cells at the base of the crypts (Figure 5c).

There were no gross or microscopic retinal lesions, but there was patchy discontinuous mineralization of the scleral collagen surrounding the tapetal choroid.

### 2.4. Genetic Analysis

Whole genome sequencing and variant discovery were performed using DNA isolated from the pony’s whole blood. Whole genome sequencing was performed using Illumina Novaseq (10.3X coverage). The whole genome sequence was mapped to the EquCab3.0 reference genome, and variants were identified using the whole animal genome sequencing (WAGS) pipeline [14]. Briefly, the FASTQ quality was determined using FASTQC, and adapters and duplicates were marked and removed. Files were mapped to the EquCab3.0 reference genome using BWA with base quality score recalibration. GATK HaplotypeCaller with hard filtering was used for variant identification, and joint calling occurred across a population of 1159 horses [15]. Variant annotation was performed using Ensembl VEP using the EquCab3.0 annotation with the Y chromosome [16].

BCFtools was used to extract (1) variants that were unique to the pony (heterozygous/homozygous) and absent in the other 1158 horses, (2) variants that were homozygous in the pony and not homozygous in the 1158 horses, and (3) variants that were present in the pony and present in the 1158 horses at an allele frequency of < 5%. The Ensembl VEP filter was used to remove variants that were more than 1000 bp downstream of a gene. One hundred and sixty‐nine candidate genes were identified using a keyword search for “Gardner syndrome” in Phenolyzer [17]. Variants within candidate genes were extracted using R.

High‐ and moderate‐impact variants for each VCF were extracted using Ensembl VEP [15]. Genotype accuracy was manually checked using the Integrative Genomics Viewer [18]. The variant in the *APC* gene was further investigated using multispecies alignment and ConSurf to determine the conservation score of the variant site [19]. MutationTaster was used to predict the consequence of the *APC* variant on the human protein [20].

#### 2.4.1. Variants Unique to the Pony

**Figure 6:**
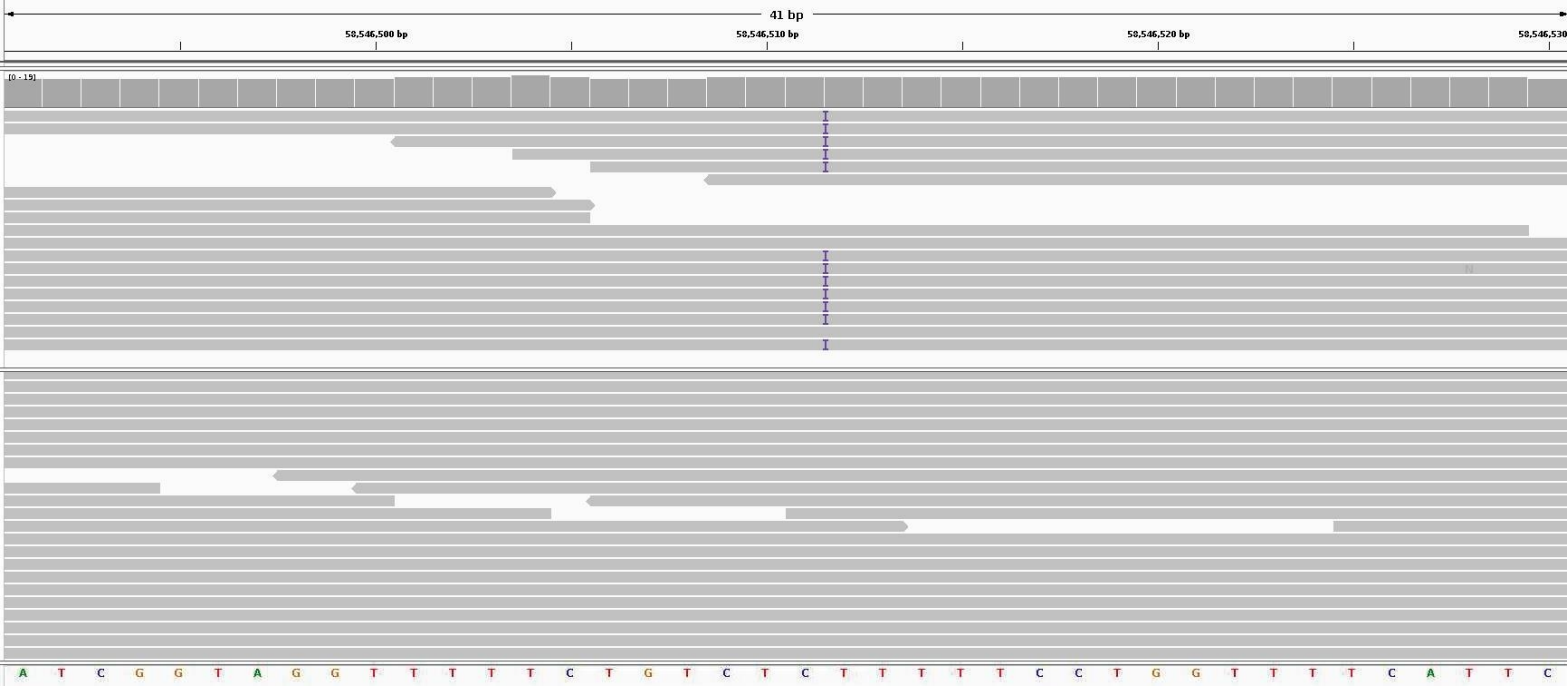
Integrative Genomics Viewer visualization of the insertion in the pony (purple vertical lines) compared to a normal control (lower sequence).

**Figure 7:**
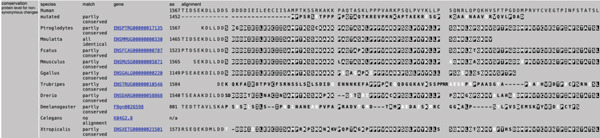
Predicted consequence of the *APC* frameshift variant and comparison of amino acid conservation across species. Photo taken from the MutationTaster prediction tool.

There were 97 high‐ and 442 moderate‐impact variants that were unique to the pony. High‐ and moderate‐impact variants were manually checked using the Integrative Genomics Viewer (Figure 6). One high‐ and five moderate‐impact variants were present in five candidate genes (Table 1). All six variants were heterozygous in the pony and absent in the 1158 horses. The high‐impact variant was present in *APC* (ENSECAP00000007276.1:p.Glu1527ArgfsTer9). This variant was a C>CT variation that was predicted to lead to a frameshift and a premature stop codon nine amino acids downstream. MutationTaster also predicted nonsense‐mediated decay and a premature stop codon nine amino acids downstream of the variant (Figure 7), leading to a truncation of the protein at Position 1534, compared to the typical protein length of 2819 amino acids.

**Table 1:** Variants that were unique to the pony.

Chrom	Position	Ref	Alt	Consequence	Impact	Gene	Exon	HGVSc	HGVSp	GT	Gene rank	HI	GI	IGV
14	58546511	C	CT	Frameshift	High	*APC*	15/15	c.4578dup	p.Glu1527ArgfsTer9	0/1	1	0.47	0.99	Yes
16	54876440	A	AC	Frameshift	High	*TGFBR2*	6/10	c.935_936insG	p.Ala313CysfsTer29	0|1	69	0.97	0.50	No
16	54876442	G	A	Missense	Moderate	*TGFBR2*	6/10	c.934C>T	p.Leu312Phe	0|1	69	0.97	0.50	No
16	54876443	G	T	Missense	Moderate	*TGFBR2*	6/10	c.933C>A	p.His311Gln	0|1	69	0.97	0.50	No
16	54876445	GC	G	Frameshift	High	*TGFBR2*	6/10	c.930del	p.Glu310AspfsTer50	0|1	69	0.97	0.50	No
16	54876455	C	A	Missense	Moderate	*TGFBR2*	6/10	c.921G>T	p.Glu307Asp	0|1	69	0.97	0.50	No
16	54876480	G	C	Missense	Moderate	*TGFBR2*	6/10	c.896C>G	p.Thr299Ser	0|1	69	0.97	0.50	Yes
24	8846353	A	T	Missense	Moderate	*HIF1A*	11/17	c.1556A>T	p.Glu519Val	0|1	96	0.98	0.61	Yes
10	12023373	G	A	Missense	Moderate	*TGFB1*	6/7	c.1028C>T	p.Ala343Val	0|1	129	0.75	0.55	Yes
10	12023385	G	C	Missense	Moderate	*TGFB1*	6/7	c.1016C>G	p.Ala339Gly	0|1	129	0.75	0.55	No
10	12023387	G	T	Missense	Moderate	*TGFB1*	6/7	c.1014C>A	p.Phe338Leu	0|1	129	0.75	0.55	Yes
29	27188227	C	T	Missense	Moderate	*GATA3*	3/6	c.313G>A	p.Gly105Ser	0|1	133	0.62	0.84	Yes

Abbreviations: Alt, alternate allele; Chrom, chromosome; GI, gene intolerance score; GT, genotype; HI, haploinsufficiency score; IGV, Integrative Genomics Viewer interpretation of true variant (yes/no); Ref, reference allele.

#### 2.4.2. Variants Homozygous in the Pony and Heterozygous or Wild Type in the Equine Population

There were two high‐ and 22 moderate‐impact variants that were homozygous in the pony and only heterozygous or wild type in the 1158 horses. None of these variants were present in candidate genes.

#### 2.4.3. Variants Present in the Pony and Rare (Allele Frequency < 0.05) in the Equine Population

There were 693 high‐ and 5690 moderate‐impact variants that were present in the pony and present in the 1158 horses at an allele frequency of < 0.05. High‐ and moderate‐impact variants were manually checked using the Integrative Genomics Viewer. One high‐ and 24 moderate‐impact variants were present in 25 candidate genes (Table 2). The pony was homozygous for two of these variants and heterozygous for the remaining 24 variants.

**Table 2:** Variants that were present in the pony and in the 1158‐horse population at a frequency of < 0.05.

Chrom	Position	Ref	Alt	Consequence	Impact	Gene	Exon	HGVSc	HGVSp	GT	Gene rank	HI	GI	IGV
9	62270787	A	G	Start lost	High	*EIF3H*	1/9	c.2T>C	p.Met1?	0/1	93	0.97	0.73	Yes
1	75700664	C	T	Missense	Moderate	*MTR*	31/36	c.3023G>A	p.Arg1008Gln	0/1	55	0.64	0.30	Yes
1	75760011	T	C	Missense & splice region	Moderate	*MTR*	13/36	c.929A>G	p.Asp310Gly	0/1	55	0.64	0.30	Yes
1	120151223	G	C	Missense	Moderate	*CYP1A1*	2/7	c.156G>C	p.Leu52Phe	0/1	29	0.08	0.16	Yes
10	4379823	C	T	Missense & splice region	Moderate	*GPATCH1*	6/20	c.535C>T	p.Pro179Ser	0/1	117	0.10	0.62	Yes
10	12023379	A	G	Missense	Moderate	*TGFB1*	6/7	c.1022T>C	p.Ile341Thr	0|1	129	0.75	0.55	Yes
11	15494088	C	T	Missense	Moderate	*GH1*	3/5	c.175C>T	p.Arg59Cys	0/1	98	0.09	0.27	Yes
11	50378461	A	G	Missense	Moderate	*ALOX12*	10/14	c.1369A>G	p.Ser457Gly	0/1	35	0.22	0.12	Yes
15	60738448	G	C	Missense	Moderate	*CYP1B1*	4/4	c.1344G>C	p.Lys448Asn	0/1	41	0.09	0.22	Yes
16	24594804	G	A	Missense	Moderate	*LRIG1*	8/19	c.1084G>A	p.Val362Ile	0/1	126	0.64	0.26	Yes
16	49673977	A	C	Missense	Moderate	*MLH1*	12/18	c.1074T>G	p.Asp358Glu	1/1	47	0.73	0.20	Yes
17	71503302	C	T	Missense	Moderate	*SLC10A2*	1/6	c.94G>A	p.Val32Met	0/1	73	0.23	0.54	Yes
17	77140780	C	G	Missense	Moderate	*IRS2*	1/2	c.3322G>C	p.Val1108Leu	0/1	94	0.61	0.00	Yes
18	68061042	G	A	Missense	Moderate	*NABP1*	6/8	c.496G>A	p.Ala166Thr	0/1	118	0.00	0.67	Yes
2	40068734	A	G	Missense	Moderate	*MTHFR*	12/13	c.2009A>G	p.Asn670Ser	0/1	19	0.12	0.39	Yes
2	73347889	C	T	Missense	Moderate	*FSTL5*	14/16	c.1624C>T	p.Pro542Ser	0/1	137	0.15	0.19	Yes
22	35840790	C	A	Missense	Moderate	*MMP9*	12/15	:c.1715C>A	:p.Pro572His	0/1	54	0.22	0.15	Yes
22	42353076	T	C	Missense	Moderate	*CYP24A1*	8/12	c.1117A>G	p.Asn373Asp	0/1	141	0.08	0.44	Yes
3	3707492	A	G	Missense	Moderate	*NOD2*	4/11	c.1754A>G	p.Gln585Arg	0/1	106	0.12	0.18	Yes
3	8353934	T	TGGCACC	Missense	Moderate	*MMP2*	9/13	c.1207_1212dup	p.Thr403_Gly404dup	1/1	26	0.88	0.51	Yes
3	79259453	A	G	Missense	Moderate	*KDR*	13/30	c.1807A>G	p.Met603Val	0/1	74	0.40	0.94	Yes
30	12166608	C	T	Missense	Moderate	*DUSP10*	3/5	c.106G>A	p.Ala36Thr	0|1	147	0.69	0.77	Yes
31	8077948	T	C	Missense	Moderate	*SLC22A3*	3/10	c.715A>G	p.Ile239Val	0/1	134	0.09	0.76	Yes
5	6150612	C	T	Missense	Moderate	*SELE*	7/12	c.1160G>A	p.Arg387His	0/1	86	0.07	0.06	Yes
8	32210658	T	C	Missense & splice region	Moderate	*POLE*	34/48	c.4402A>G	p.Ser1468Gly	0/1	18	0.63	0.88	Yes

Abbreviations: Alt, alternate allele; Chrom, chromosome; GI, gene intolerance score; GT, genotype; HI, haploinsufficiency score; IGV, Integrative Genomics Viewer interpretation of true variant (yes/no); Ref, reference allele.

## 3. Discussion

FAP is a rare hereditary condition, which is estimated to affect approximately 1 in 8000 people with no sex predilection [1–4, 21]. In its classic form, it is characterized by numerous adenomatous polyps in the colon. A variety of extracolonic manifestations can accompany the polyps, and these define various subtypes within the spectrum of FAP [1–4, 10, 21, 22]. While Gardner syndrome has historically been considered a different genetic condition from FAP, it is now considered a variant or subtype of FAP, which exists on a spectrum of clinical manifestations. The extracolonic manifestations of Gardner syndrome include osteomas, epidermoid cysts, dental abnormalities, dermoid tumors, and CHRPE [1–4]. In human patients, the intestinal polyps most often begin to develop during early adolescence and increase with age. Without surgical intervention, the lesions almost invariably progress to colorectal cancer by around the age of 40. While there is no cure for FAP, early identification and monitoring play a crucial role in the management of the condition.

FAP is an autosomal dominant condition caused by a germline mutation in the APC gene [5–9, 23, 24]. While most affected individuals have a family history of FAP, in approximately 20%–30% of patients, a family history is lacking, indicating a de novo mutation in the APC gene. The p.Glu1527ArgfsTer9 variant identified in this pony leads to an altered amino acid sequence from **E**TEKPTDSE (reference) to **E**DRKTYRF∗ (variant) and a premature stop codon at Position 1534 compared to the reference APC protein length of 2819 amino acids. It is not possible to comment on the inheritance of this variant because the parents of this pony are not known. As the pony is heterozygous for this variant, it would suggest that if this is the disease‐causing variant, it follows a dominant inheritance pattern.

The *APC* gene encodes the APC tumor suppressor protein that performs diverse cellular functions, including the regulation of *β*‐catenin, a widely expressed protein involved in the regulation and coordination of cell–cell adhesion and gene transcription [5–9, 23, 24]. When APC function is compromised, there is constitutive activation of *β*‐catenin, resulting in the loss of cell cycle checkpoints and unregulated cellular proliferation. Most of the variants reported to be associated with colorectal cancer occur in the “mutation cluster region,” including Amino Acids 1284–1580. The mutation cluster region is in the middle of the *APC* gene and is critical for the assembly of the *β*‐catenin destruction complex. This variant identified in this pony maps to Amino Acid Position 1552 in the human *APC* gene, which is within the mutation cluster region. The same variant in the human genome would be predicted to lead to EAEKTIDSE (reference) to EGRKNY∗ (variant) and a premature stop codon at Position 1557 compared to the reference APC protein length of 2843 amino acids. There are four variants identified in patients with FAP reported in ClinVar that affect the glutamic acid at Position 1552 in the human *APC* gene: two frameshift variants (p.Glu1552fs), one premature stop codon variant (p.Glu1552Ter), and one synonymous variant (p.Glu1552=). Except for the synonymous variant that does not change the glutamic acid, the variants have sufficient evidence to be pathogenic or pathogenic/likely pathogenic. In the region of the protein affected by this mutation (from Positions 1552 to 2843), there are 8419 mutations, of which 451 are frameshift or premature stop codon–inducing variants. Seven of the 451 variants have conflicting evidence of pathogenicity, and 18 are considered variants of uncertain significance. These variants are all at Position 2655 or further toward the end of the protein. The remaining 426 frameshift and premature stop codon–inducing variants are considered likely pathogenic (*n* = 20), pathogenic (*n* = 333), and pathogenic/likely pathogenic (*n* = 73). Based on the American College of Medical Genetics and Genomics and the Association for Molecular Pathology guidelines, this variant has multiple levels of evidence to support it being likely pathogenic: it is a predicted null variant in a gene where loss of function is a mechanism of disease (PVS1), the variant is absent from the general population of horses (PM2), and multiple lines of computational evidence support a deleterious effect on the gene product (PP3).

The only other high‐impact variant that was present in a candidate gene and appeared to be real based on visualization was a p.Met1? variant in *EIF3H*. This gene is known to be a cause of hereditary colorectal cancer in a recessive inheritance pattern. However, there has not yet been an association with the generalized osteomas seen in this pony. The pony was heterozygous for this variant, and the variant was present in the general population of 1158 horses at an allele frequency of 0.009. There were 18 alleles in this population, with 16 other horses being heterozygous for the variant and one being homozygous. Although we do not have phenotypes on these horses, given the rarity of the affected pony’s clinical signs, it is unlikely that this variant is causing this disease.

Gardner syndrome, now recognized as a subtype of FAP, was first described in 1951 by Eldon J. Gardner [25] as a genetic condition associated with a cluster of cases of carcinoma of the lower digestive tract. Gardner syndrome is characterized by intestinal polyposis and a variety of extracolonic abnormalities, most often including multiple osteomas, especially involving the skull (maxillofacial) and various soft tissue tumors, including epidermal cysts and desmoid tumors [1–4, 21, 22, 26]. Less common extracolonic manifestations include abnormal dentition, congenital hypertrophy of the retinal pigmented epithelium, adrenocortical adenoma and carcinoma, and a variety of other neoplasms. The occurrence of the extracolonic manifestations seen in Gardner syndrome appears to be determined by the location of the defect on the APC gene and epigenetic factors.

This pony had multiple osteomas (especially severe involvement of the skull), intestinal mucosal polyps, epidermal inclusion cysts, dental disease, and adrenal adenomas, all of which are components of Gardner syndrome in humans. Osteomas have been infrequently reported in horses, most often involving the craniofacial complex [27–41]. However, unlike the osteomas in this case, they are typically isolated lesions. Polyps of the gastrointestinal tract are rarely reported in horses [42–44, 42, 43, 45, 46]. This pony had dozens of intestinal mucosal polyps that were limited to the small intestine, and no malignant progression was identified. In human patients with Gardner syndrome, the colon and rectum are most often affected, but upper gastrointestinal polyps and carcinoma have been identified [3, 47]. Skin and adrenocortical tumors similar to those in humans with Gardner syndrome were also present in this pony. Congenital hypertrophy of the retinal pigmented epithelium as seen in Gardner syndrome was not identified in this pony, although mineralization of the sclera surrounding the tapetum was present bilaterally. In human patients with Gardner syndrome, the exact signs vary between individual patients.

This patient presented with dental disease and nasal discharge, which was suspected to be due to secondary sinusitis. In general, apical infections are a common cause of sinusitis in the horse due to the close proximity of the apices of the upper cheek teeth to the sinus and nasal cavities, allowing for contamination of these structures by bacteria [48, 49]. However, this case was unique in that the severe dental disease was associated with extensive osteoma formation throughout the mandible and maxilla, including the sinus compartments. The presence of osteomas within the sinus compartments resulted in obstruction of the normal outflow tracts, resulting in bilateral mucopurulent nasal discharge as well as malformation of the patient’s skull and misalignment of the teeth. In people with FAP, the reported prevalence of dental abnormalities varies, ranging from 30% to 75% of patients [21, 50–54]. Importantly, the dental manifestations may precede the development of intestinal polyposis, making identification of the dental features of Gardner syndrome helpful in the early diagnosis of the condition.

FAP and its variants, including Gardner syndrome, have not been reported outside of human patients. The severe dental disease and multiple bony enlargements in this pony were suggestive of Gardner syndrome, and this was further supported by diagnostic imaging, biopsies, and necropsy. Although it can be challenging to interpret genetic analysis from a single patient, the heterozygous single base pair insertion variant leading to a frameshift in the *APC* gene is similar to human variants considered pathogenic or likely pathogenic for FAP in the ClinVar database [55]. Further investigation of this variant is necessary to confirm that this is the causative variant in this case. Additionally, while unable to be performed at this time, genome sequencing of the intestinal polyp to identify the APC mutation would have strengthened the case for FAP. Overall, the findings in this patient are highly suggestive of Gardner‐like syndrome with a suggested genetic cause, making it the first known reported case in a veterinary species.

## Funding

Funding was provided by the National Institutes of Health (10.13039/100000002) under grant numbers K12TR002492 and UL1TR002494.

## Conflicts of Interest

The authors declare no conflicts of interest.

## Data Availability

Data are available on request from the authors.
